# Does artemether–lumefantrine administration affect mosquito olfactory behaviour and fitness?

**DOI:** 10.1186/s12936-019-2646-9

**Published:** 2019-01-28

**Authors:** Jetske G. de Boer, Annette O. Busula, Jet ten Berge, Tessa S. van Dijk, Willem Takken

**Affiliations:** 1 Laboratory of Entomology, Wageningen University & Research, Droevendaalsesteeg 1, 6708 PB Wageningen, The Netherlands; 2 Netherlands Institute of Ecology, Droevendaalsesteeg 10, 6708 PB Wageningen, The Netherlands; 3 International Centre of Insect Physiology and Ecology, P.O BOX 30772-00100 GPO, Nairobi, Kenya; 4 Present Address: Kaimosi Friends University College, P.O BOX 385-50309, Kaimosi, Kenya

**Keywords:** Skin odour, Olfaction, Host-searching, Antimalarial medication, Epidemiology, Post-treatment transmission, Gametocytes

## Abstract

**Background:**

Artemisinin-based combination therapy (ACT) is the recommended treatment against uncomplicated *Plasmodium falciparum* infections, and ACT is widely used. It has been shown that gametocytes may be present after ACT and transmission to mosquitoes is still possible. Artemether–lumefantrine (AL) is a broadly used artemisinin-based combination medicine. Here, it is tested whether AL influences behaviour and fitness of *Anopheles* mosquitoes, which are the main vectors of *P. falciparum*.

**Results:**

Dual-choice olfactometer and screenhouse experiments showed that skin odour of healthy human individuals obtained before, during and after AL-administration was equally attractive to *Anopheles coluzzii* and *Anopheles gambiae* sensu stricto, apart from a small (but significant) increase in mosquito response to skin odour collected 3 weeks after AL-administration. *Anopheles coluzzii* females fed on parasite-free blood supplemented with AL or on control-blood had similar survival, time until oviposition and number of eggs produced.

**Conclusions:**

Based on the results, AL does not appear to influence malaria transmission through modification of vector mosquito olfactory behaviour or fitness. Extending these studies to *Plasmodium*-infected individuals and malaria mosquitoes with parasites are needed to further support this conclusion.

**Electronic supplementary material:**

The online version of this article (10.1186/s12936-019-2646-9) contains supplementary material, which is available to authorized users.

## Background

Artemisinin-based combination therapy (ACT) has been the recommended treatment against uncomplicated malaria caused by *Plasmodium falciparum* since 2002 [1]. It is also often used against other *Plasmodium* species. In 2016, over 400 million doses were distributed, with the large majority being administered in Africa [2]. ACT has made a significant contribution to the reduction in malaria cases observed between 2000 and 2015, supporting their important role in malaria control [3]. ACT quickly reduces malaria symptoms and densities of asexual malaria parasites [4], while it can also reduce gametocyte carriage and malaria transmissibility compared to non-ACT medicines [5–8]. A recent meta-analysis concluded that the most widely used ACT medicine, artemether–lumefantrine (AL), is superior to non-ACT medicines in reducing gametocytaemia and malaria transmission [9]. Nevertheless, ACT has a limited effect against mature gametocytes, and gametocytes can still be transmitted from *P. falciparum*-infected patients following ACT [10–14].

Gametocytes are transmitted to mosquito females when they take a blood meal from infectious humans, and, after parasite development in infected mosquitoes, malaria parasites are transmitted to humans in the form of sporozoites, again during blood feeding. *Anopheles gambiae* sensu lato (s.l.). and *Anopheles funestus* s.l. are the most important vector species in sub-Saharan Africa [15], partially due to their strong preference for blood feeding on humans [16]. To find a host, mosquito females use volatile cues emanating from the skin, besides CO_2_ that is associated with the presence of all vertebrate hosts. In *An. gambiae* sensu stricto (s.s.) and *Anopheles coluzzii*, siblings within the *An. gambiae* species complex, anthropophilic biting behaviour is associated with a strong preference for human skin odour over skin odour of other vertebrate host species [e.g. 17].

Surprisingly, there are no studies on the possible effects of ACT on malaria-transmitting mosquitoes. Yet, it is important to investigate such effects because transmission of gametocytes to mosquitoes is still possible after ACT as described above [e.g. 14]. The rate of post-treatment transmission could be influenced if ACT has an effect on the attractiveness of treated individuals and consequently on the probability of contact between ACT-treated humans and mosquitoes. Several factors are known to affect human attractiveness to mosquitoes through effects on skin odour composition, including infection with malaria parasites [18]. Here, the effect of AL on the attractiveness of parasite-free humans is assessed by studying mosquito responses towards their skin odour.

The rate of malaria parasite transmission could also be affected by ACT, if ACT would directly affect the lifespan of infected mosquitoes and consequently the probability of parasites within the mosquito reaching the sporozoite stage. Female mosquito fitness was therefore studied when females take a blood-meal supplemented with AL. Although *Plasmodium* infection may affect mosquito survival [e.g. 19], and the effects of ACT may be different in infected mosquitoes, uninfected mosquitoes were used in this study as a first investigation of the potential endectocidal effects of ACT against *Anopheles*.

## Methods

### Mosquitoes

The mosquitoes used for the laboratory experiments in Wageningen, The Netherlands, were from a colony of *An. coluzzii* [20], which originated from Suakoko, Liberia. The colony has been reared in the laboratory since 1987 according to methods described by de Boer et al. [21], and membrane-fed on human blood (Sanquin Blood Supply Foundation, Nijmegen, The Netherlands) in the presence of human odour (from a worn sock) and 5% CO_2_. Newly emerged mosquitoes were supplied with 6% glucose but no blood prior to experiments. Female mosquitoes were approximately 7 days old when they were used in the fitness experiment, and approximately 8 days old in the olfactometer experiment. Experimental mosquitoes were selected in the afternoon prior to the start of an experiment and provided with water only until use.

The semi-field experiments were performed at the Thomas Odhiambo Campus of the International Centre of Insect Physiology and Ecology (ICIPE) in Mbita, western Kenya. A laboratory colony of *An. gambiae* s.s., originating from Mbita and kept in the laboratory since 2001, was reared according to methods described by Busula et al. [17] and fed three times per week on a human arm. Experimental mosquitoes (2 to 7 days old) were collected in the morning, and kept with water only until the start of an experiment the same evening.

### Effect of AL on attractiveness of human skin odour to mosquitoes

#### Collection of odour samples

**Fig. 1:**
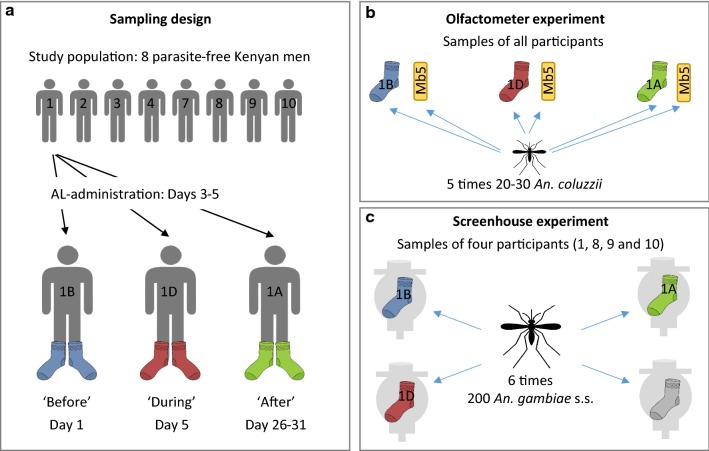
Sampling and experimental design of mosquito attraction to skin odour of participants to whom AL was administered. **a** Eight parasite-free Kenyan men (20–40 years old) were provided with six doses of AL over 3 days. Worn nylon socks were collected before, during and after AL-administration to obtain skin odour samples for mosquito experiments. **b** Skin odour samples were tested for relative attractiveness in a dual-port olfactometer with *An. coluzzii*. Each sample was tested against a control synthetic odour blend Mb5. **c** A direct comparison of attractiveness of skin odour samples was made in a screenhouse with *An. gambiae* s.s. using MM-X traps

The samples used for the attractiveness experiments were collected from eight non-smoking Kenyan men between 20 and 40 years old. Their blood was tested for the presence of *Plasmodium* parasites through RDT and nested PCR on a fragment of the 18S rRNA gene [22]. Individuals with an in-ear temperature higher than 37.5 °C, or carrying *Plasmodium* detected by either diagnostic method were excluded. Participants were asked not to use alcohol and spicy food for 24 h before, and during sample collection. They were also asked to take their last shower before sample collection without soap, and not to shower during sample collection. Odour samples were obtained by wearing a pair of nylon socks (97% polyamide, 3% elastane, 20 denier, HEMA, The Netherlands) for approximately 20 h. Nylon socks were washed with 70% EtOH before use and dried in an oven at 70 °C for 2 h. ‘Before’ samples were collected on day 1 of the trial. On day 3, the participants started with a 3-day course of anti-malarial treatment at the same dose and for the same duration as recommended for uncomplicated malaria, i.e. six doses of four tablets containing 20 mg artemether and 120 mg lumefantrine per tablet at intervals over 3 days [18]. ‘During’ samples were collected on day 5 (i.e. on the final day of anti-malarial treatment) and ‘after’ samples were collected between day 26 and 31 of the trial (Fig. 1). Artemether and lumefantrine have a half-life of about 2 h and 3–6 days, respectively [23, 24], so 3 weeks should be sufficient to ensure that only small amounts of lumefantrine (between approximately 0.5 to 5%) and no artemether are left in the body. A new pair of socks was used for each sample. The odour samples were stored in the freezer at -20 °C until use and between experiments. Surgical gloves were worn during all handling to avoid contamination with human skin odour. Ethical approval for this study was obtained from the Kenyan Medical Research Institute (Non-SSC protocol 389) and all participants provided informed signed consent.

#### Olfactometer

The attractiveness of odour samples of all participants was tested in a dual-port olfactometer with three identical flight chambers (Tupola, The Netherlands), according to methods described by Verhulst et al. [25]. Before use in the olfactometer, the foot-part was removed from one worn sock of each sample. Half of the remaining leg-part (approximately 30 by 5 cm) was used as the test odour source in the olfactometer trap. The other trap contained a nylon strip (26.5 × 5 cm) baited with the Mb5 blend—a synthetic human odour mimic [26]—to obtain a relative measure of attractiveness of each skin odour sample (Fig. 1). Charcoal-filtered, moist and heated air (23.9–32.8 °C [95% confidence interval, CI]) was blown through the two trapping devices at a wind speed of 0.2 ± 0.05 m/s. Temperature inside the flight chambers was 25.1–28.3 °C (95% CI), and humidity was 55–74% (95% CI). The experimental room was heated to 25.6–28.4 °C (95% CI) and had a relative humidity of 39–55% (95% CI). CO_2_ (5%) was provided at approximately 175 mL/min at 5 cm below each trap entrance.

Each sample was tested once in five of the six positions of the olfactometer over five different days to obtain a thorough assessment of its attractiveness. In every test, 20 female *An. coluzzii* were used, with the exception of the first replicate for each sample, for which 30 mosquitoes were released. The eight participants were divided in two groups, which were tested on different days, with the three sample time points per participant kept together in one group. Each Mb5-baited strip was used in only one dual-choice test per day, and stored at 4 °C between experimental days.

#### Semi-field experiment

Skin odour samples collected from four randomly selected participants were used to directly compare attractiveness of samples obtained before, during and after AL-administration per participant. Experiments were conducted as described before [17, 27], in two greenhouses (7 × 11 × 2.5 m) with glass-panelled roofs and gauze-covered sidewalls with sand on the floor. MM-X traps (American Biophysics Corp., North Kingstown, USA) were placed in each corner and baited with a nylon sock of each of the three sampling moments or a new unworn sterile nylon sock as a control (Fig. 1). The traps were each assigned to a specific treatment. The MM-X traps were provided with CO_2_ from fermented sugar [17, 27]. Each test started with trapping devices cleaned with 70% EtOH, and new mosquitoes were used for every experiment. The greenhouse was set for the experiment 2–5 h before the start by removing remaining mosquitoes, spiders, spider webs and by moistening the sand in the greenhouse. Two hundred female *An. gambiae* s.s. mosquitoes were released from a paper cup at the centre of the greenhouse at 20.00 h. In the morning (7.00 h), the traps were collected, placed in a freezer and trapped mosquitoes counted. Samples of the four participants were each tested six times, and participants were alternated between the testing nights. Within participants and between replicates, the traps were randomly rotated among the different corners of the greenhouse.

### Effect of ACT on mosquito fecundity and survival

One day before the start of the experiment, two groups of forty *An. coluzzii* females were placed inside mesh cages (15 × 15 × 15 cm), and provided with tap water on cotton wool. The next morning, during the last 3 h of the scotophase, a membrane with 1 mL human blood was placed on top of each cage. In the AL-treatment group, the membrane contained blood to which dissolved AL was added. In our in vitro assay, we used the concentration of one dose of AL (4 times 20 mg artemether and 120 mg lumefantrine) in the volume of blood of an adult person (6 L). This concentration is 10–100 times higher than peak levels reached in vivo, which also depend on food intake and presence of *Plasmodium* parasites [24, 28, 29]. In vivo, artemether and lumefantrine also have different ratios because of differences in half-life and they are metabolized into different active compounds in the human body. A suspension of an AL tablet was made in PBS at a concentration of 0.29 mg/mL artemether and 1.71 mg/mL lumefantrine. Fifty µl of this suspension was added to 950 µL blood resulting in a concentration of 13.3 µg/mL artemether and 80 µg/mL lumefantrine. The control group received 950 μL blood to which 50 μL PBS was added. A few hours after feeding, all visibly blood-fed mosquitoes were photographed using a camera mounted on a stereomicroscope to measure wing length, using ImageJ software. To immobilize mosquitoes, they were placed in glass vials on ice for about 30 s and then transferred to a Petri dish with ice, placed under the microscope. Mosquitoes were then placed individually in a paper coffee cup (150 mL), which contained a smaller plastic medicine cup (25 mL) filled with tap water and a cone-shaped filter paper (diameter 9 cm) for oviposition. Paper cups were covered with mesh and 6% glucose solution was provided on cotton wool on top of the mesh. Experimental mosquitoes were kept in a climate room under the same conditions as the colony. Survival and oviposition were monitored daily for 14 days to record the day that the first eggs were laid and the day of death. When eggs were found, the filter paper was replaced and the eggs were counted using a stereomicroscope. Three experimental series were done with approximately 40 mosquitoes per group per series.

### Statistical analysis

#### Olfactometer experiment

Mosquitoes caught on the skin odour samples and controls were summed over the five testing days before statistical analyses, accounting for the non-independence of the repeated attractiveness measures per sample. Two parameters were defined to evaluate the effect of AL-administration. Relative attractiveness was calculated as the mosquitoes caught in the trap baited with the skin odour sample as a proportion of the total number of mosquitoes caught in both traps (i.e. caught on the sock and on the Mb5-baited strip). Mosquito response was calculated as the number of mosquitoes caught in the trap baited with the skin odour sample divided by the number of mosquitoes that left the release cage in that particular test, i.e. irrespective of the mosquitoes caught on the control odour. Generalized linear models (GLM, Binomial distribution, logit link function, deviation estimated) were used to statistically analyse the effect of AL-administration on these parameters separately. The number of mosquitoes caught in the trap with the skin odour sample was used as the response variable in both models, while binomial totals were the total number of mosquitoes in both traps for relative attractiveness, and the number of mosquitoes that left the release cage for mosquito response. Participant identity was included in the models to account for variation in intrinsic attractiveness between individuals [25]. Differences between sample time points were tested by pairwise comparisons using least significant differences at the 5% level (LSD). Relative attractiveness of each sample was also evaluated with Binomial tests on the numbers of mosquitoes caught on the skin odour sample or on the control, and comparing it to 0.5.

#### Screenhouse experiment

A binomial GLM was used to evaluate the effects of treatment (sampling time point relative to AL-administration or control odour), participant and their interaction on the proportion of mosquitoes trapped by the different odour samples. The number of mosquitoes per trap was used as the response variable and the total number of mosquitoes trapped per experimental night per screenhouse was used as the binomial total. Position of the trap within the screenhouse was also fitted in the GLM because it had a significant effect on mosquito catches.

#### Mosquito fitness

Mosquito survival after blood-feeding and time until oviposition were compared between the AL and control group with a Kaplan–Meier survival test using the long-rank Mantel-Cox χ^2^ statistic. Only mosquitoes that laid eggs were included in the analysis of time until oviposition. To examine if AL affects the proportion of blood-fed mosquitoes that laid eggs, Fisher’s exact probability test (2 × 2 contingency table) was used. A Poisson GLM (log-link function) was used to test the effect of AL on the number of eggs produced. Treatment, series and the interaction were included in the model, and wing length was used as a covariate because mosquito fitness is known to be associated with size [30]. Non-significant (P > 0.05) terms were dropped from the final model. Statistical analyses were done with SPSS software, version 24 (IBM Corp.).

## Results

### Effect of anti-malarial administration on mosquito olfactory behaviour in the dual-choice olfactometer

**Fig. 2:**
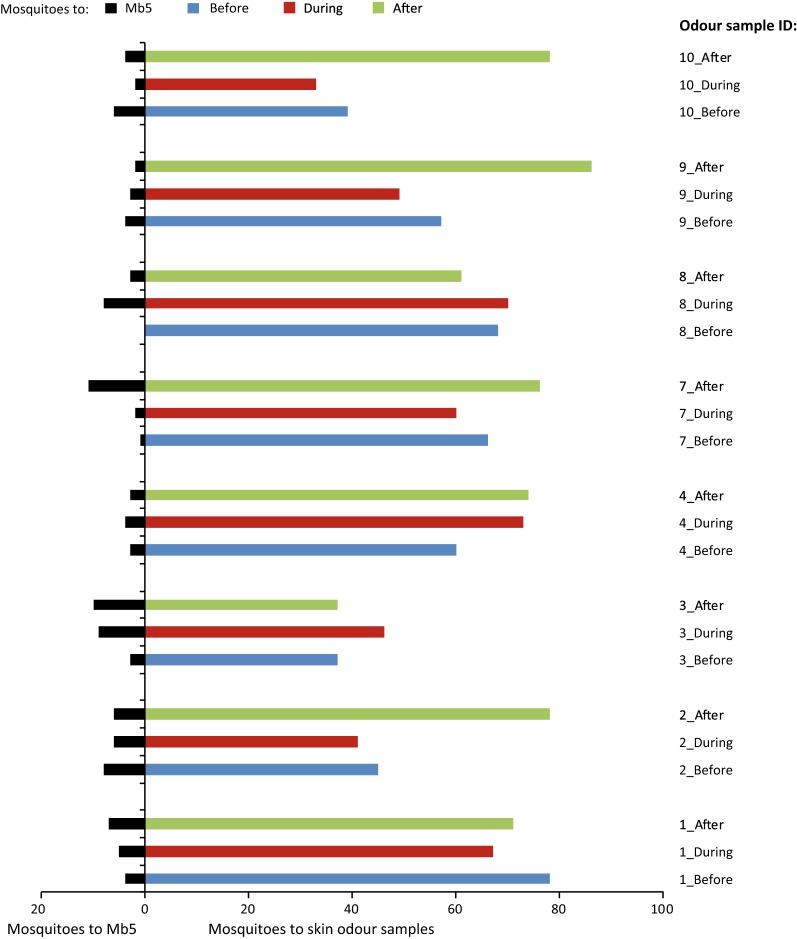
Attractiveness to *Anopheles coluzzii* mosquitoes of skin odour samples of eight parasite-free participants to whom AL was administered. Skin odour samples were collected at three time points relative to AL-adminstration, i.e. before, during and after. Each sample was tested against a synthetic odour bait (Mb5) in a dual-port olfactometer. Each sample was tested five times with 20–30 mosquitoes on five different days for a total of 110 mosquitoes per samples. Numbers of mosquitoes attracted to skin odour samples and Mb5-control are summed over these five times. In all cases, mosquitoes significantly preferred skin odour to Mb5 control (Binomial tests, P < 0.001). See Additional file 2 for data

**Fig. 3:**
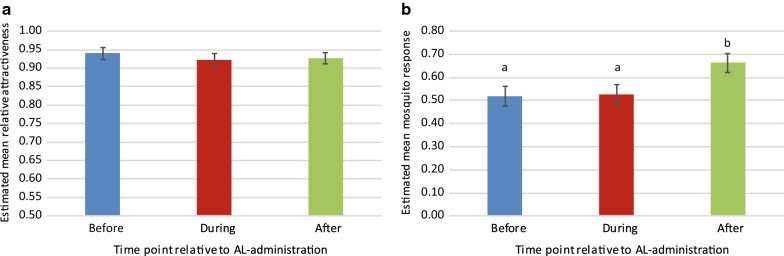
Effect of AL-administration on attractiveness of skin odour to *Anopheles coluzzii* in a dual-port olfactometer. Attractiveness of skin odour samples obtained from eight participants at three time points relative to AL-administration (before, during, after) was tested, with five replicates of 20–30 mosquitoes per skin odour sample. **a** Relative attractiveness reflects the percentage of mosquitoes that were trapped on human skin odour samples as a proportion of the total number of mosquitoes trapped on skin odour and the Mb5 control. Time point relative to AL-administration and participant identity had no significant effect on relative attractiveness (GLM, P_time point_ = 0.720; P_participant_ = 0.186). **b** Mosquito response reflects the mosquitoes trapped on human skin odour samples as a proportion of the number of mosquitoes that flew in the olfactometer, i.e. independent of the mosquitoes trapped on the Mb5 control. Time point relative to AL-administration had a significant effect on mosquito response (GLM, P_time point_ = 0.026). Different lower case letters above bars indicate pairwise significant differences (GLM, LSD, P < 0.05). Participant identity also had a significant effect on mosquito response (GLM, P_participant_ = 0.033). Estimated means with standard errors from GLMs are shown. See Additional file 2 for data

For all 24 skin odour samples (before, during or after AL-administration of each of eight participants), the worn sock attracted significantly more mosquitoes than the control Mb5 bait (Binomial tests, P < 0.001, Fig. 2). Mosquito choice for worn socks ranged between 79 and 100%, and was not affected by sampling time point, i.e. before, during or after AL-administration (GLM, P = 0.712, Fig. 3a) or participant (P = 0.186).

The mosquito response to skin odour samples was more variable, ranging from 31 to 83% of mosquitoes that flew in the olfactometer being trapped on the skin odour sample (Fig. 2). Mosquito response to worn socks was significantly affected by sampling time point relative to AL-administration (GLM, P = 0.026, Fig. 3b). Significantly more mosquitoes responded to skin odour collected after AL-administration than to skin odour collected before or during AL-administration (GLM, LSD, P = 0.014 and P = 0.020, respectively). Mosquito responses to skin odour collected before or during AL-treatment were statistically similar (GLM, LSD, P = 0.914). Participant identity had a significant effect on mosquito responses to skin odour (GLM, P = 0.033), with participant #3 attracting significantly fewer mosquitoes than most other participants, except participants #2 and #10 (Fig. 2 and Additional file 1: Table S1).

### Effect of anti-malarial administration on mosquito olfactory behaviour in a semi-field experiment

**Fig. 4:**
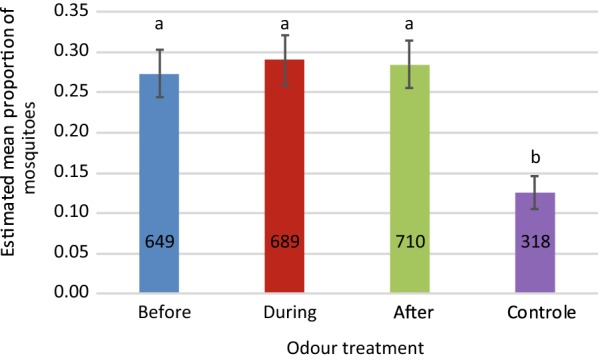
Effect of AL-administration on screenhouse catches of *Anopheles gambiae* s.s. on skin odour samples. For each of four participants, skin odour samples obtained at three time points relative to AL-administration (before, during, after) were tested for attractiveness against clean nylon socks (control) in MM-X traps. CO_2_ was added to each trap. Samples from the same participant were tested in direct competition against each other, and experiments were repeated on six different nights per participant with 200 females per night. Numbers in bars indicate the total number of mosquitoes caught per odour treatment. Odour treatment had a significant effect on the proportion mosquitoes trapped (GLM, P_treatment_ < 0.001). Different lower case letters above bars indicate pairwise significant differences (GLM, LSD, P < 0.05). Position of the trap within screenhouse had a significant effect and the nested term was included in the final model (GLM P_screenhouse(position_) < 0.001). Estimated means with standard errors from the GLM are shown. See Additional file 2 for data

Direct comparisons of mosquito attraction to skin odour collected from individual participants at different sampling time points relative to AL-administration was tested in a screenhouse. Four of the eight participants were randomly selected for this experiment, and tests for each participant were repeated on six different nights for a total of 24 experiments. Of the 4800 mosquitoes released, 2366 *An. gambiae* were caught (49%). The response of *An. gambiae* to traps baited with skin odour samples and CO_2_ was significantly higher than to the control traps that were baited with clean socks and CO_2_ (GLM, P < 0.001). Mosquitoes did not differentiate between skin odour samples collected before, during or after AL-administration (GLM, LSD, P > 0.691, Fig. 4).

### Effect of anti-malarial administration on mosquito fitness

**Fig. 5:**
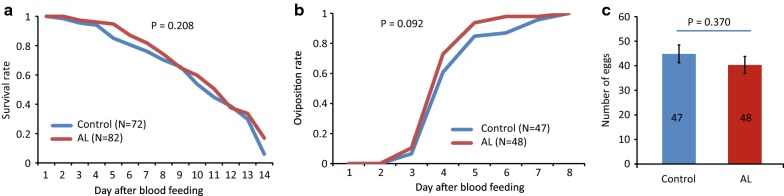
Effect of AL on fitness of *Anopheles coluzzii* mosquitoes. Mosquito females were fed on parasite-free human blood supplemented with AL or on control-blood. Survival rate (**a**) and oviposition rate (**b**) of mosquitoes after one blood meal on control-blood (blue line) or AL-blood (red line). **c** Number of eggs laid after one blood meal with AL (red) or control (blue). Number of replicates and P-values for the effect of treatment are indicated in each panel. Error bars represent standard errors of the mean. Wing length did not explain any variation in the number of eggs laid and was excluded from the final GLM. See Additional file 2 for data

Out of approximately 120 *An. coluzzii* mosquitoes that were provided with AL-blood, 82 females were considered blood-fed and were included in the fitness experiment. For the group fed on control-blood, 72 out of 120 mosquito females had blood-fed. Survival of mosquitoes fed on AL-blood and control-blood was statistically similar (Kaplan–Meier, Mantel-Cox χ^2^ = 1.582, d.f. = 1, P = 0.208, Fig. 5), with a median survival of 11 days after blood feeding for the control group and 12 days for the mosquitoes fed on AL-blood.

Feeding on AL-blood did not influence the proportion of mosquitoes that oviposited after taking one blood meal: 59% of AL-blood fed mosquitoes laid eggs, while 65% of control-blood fed mosquitoes laid eggs (Fisher exact probability test, P = 0.411). AL did not significantly affect time until oviposition with a median of four days after blood-feeding in both groups (Kaplan–Meier, Mantel-Cox χ^2^ = 2.837, d.f. = 1, P = 0.092, Fig. 5). Finally, the number of eggs laid by females fed on AL-blood (40.30 ± 3.47) was statistically similar to that of mosquito females fed on control-blood (44.83 ± 3.66) (GLM, P = 0.370, Fig. 5).

## Discussion

Malaria mosquitoes rely on skin odour to find their human host [e.g. 31], and many factors contribute to interindividual variation in attractiveness of healthy humans [25]. Here, the influence of anti-malarial treatment with ACT on human attractiveness to malaria mosquitoes was investigated. This is relevant because ACT has a limited effect against gametocytes, which can lead to post-treatment gametocytaemia and transmission to mosquitoes. A recent meta-analysis showed that approximately 25% of patients that report with gametocytes, may still carry gametocytes 1 week after AL-treatment, the most common form of ACT currently used in Africa [14]. This proportion gradually decreases over the course of the next few weeks. Several studies demonstrated that post-treatment gametocytaemia can indeed result in transmission to mosquitoes 1 week after anti-malarial treatment with ACT [13, 32]. Skin odour samples collected from healthy participants on the final day of a three-day administration course with AL had the same attractiveness as skin odour samples collected before AL-administration. This was shown in a dual-port olfactometer in the laboratory with *An. coluzzii* and in semi-field experiments with *An. gambiae* s.s., both members of the *An. gambiae* species complex. This suggests that the attractiveness of human skin odour to these malaria-transmitting mosquitoes is not altered during administration of AL to healthy persons.

Skin odour samples collected three weeks after completion of AL-administration attracted significantly more mosquitoes than skin odour samples collected before or during administration (Fig. 3b), although mosquito choice was not influenced (Fig. 3a). This effect was only observed with *An. coluzzii* in the dual-port olfactometer and not with *An. gambiae* s.s. in the semi-field experiment, perhaps due to the experimental set-up or the smaller number of participants tested in the latter experiment. To ensure that increased attraction was caused by AL-administration, and not by other factors that changed over time and influenced attractiveness to mosquitoes, e.g. climatic factors, diet or physical activity, it would have been necessary to include skin odour samples from participants that had not received AL. However, other studies suggest that human skin odour and differential attractiveness of healthy humans to mosquitoes is stable over time, although very few studies have actually tested this [33, 34]. If increased attractiveness at three weeks after AL-treatment can be confirmed in *Plasmodium*-infected patients, the question is whether this is meaningful in terms of transmission. Post-treatment gametocytaemia is also found at this time point but it occurs in less than 5% of treated patients that report with gametocytes [14], and no studies have investigated whether treated patients can infect mosquitoes at this time point. Due to this low percentage, and the relatively small increase in mosquito response to skin odour (from 52 to 66% of mosquitoes attracted), the impact of this finding on malaria transmission from humans to mosquitoes is limited at most.

Ultimately, it is essential to investigate if these findings can be translated to AL-treated *Plasmodium*-infected persons. *Plasmodium*-infection is known to influence human attractiveness to mosquitoes [35–37] through changes in skin odour profile [18], and gametocyte-infected red blood cells can emit mosquito-attractants [38]. It is possible that there are interactions between ACT and *Plasmodium*-infection that are not seen in healthy participants after administration of ACT.

Direct effects of AL on mosquitoes were also investigated by feeding females on human blood supplemented with AL. Results of the in vitro experiment suggest that uninfected *An. coluzzii* is not affected by the drug because time until oviposition, the number of eggs in the first egg batch, and survival were the same in mosquitoes fed on control- or AL-blood (Fig. 4). Based on what is currently understood about their mode of action against *Plasmodium* parasites [23, 39, 40], it was not expected that artemether and/or lumefantrine would affect fitness of *An. coluzzii*. However, differences between results in the in vitro assay and in vivo effects could arise from exposure of mosquitoes to different metabolites of artemether and lumefantrine, and different concentrations of and ratios between metabolites. In vivo, artemether and lumefantrine are quickly metabolised into dihydroartemisinin and desbutyl-lumefantrine, respectively [24, 41]. Artemether and lumefantrine have low solubility in water and due to differences in half-life [23], ratios between the metabolites would be different in vivo. Feeding mosquitoes on blood of ACT-treated patients could overcome these limitations. Moreover, effects may be different in mosquitoes that ingest ACT at the same time as gametocytes because there may be interactions between drugs and parasites. To verify this, it would be necessary to perform tests with *Plasmodium*-infected mosquitoes. Despite these limitations, our findings suggest that mosquito fitness will not be influenced by blood-feeding on ACT-treated humans, in contrast to the endectocidal drug ivermectin that proved to have a significant impact on malaria transmission through its effect on *Anopheles* mosquitoes [42].

## Conclusions

Perhaps surprisingly, there is a scarcity of studies on the effects of medication on human body odour profiles and attractiveness to mosquitoes, with the exception of a study from 1968 testing drugs and diseases as potential repellents against *Aedes* mosquitoes [43]. Here, the potential effects of AL on behaviour and fitness of malaria mosquitoes were studied. The experiments provided no evidence for a major effect of AL on human attractiveness to *An. coluzzii* and *An. gambiae* s.s., apart from a small increase in attractiveness 3 weeks after AL-administration. To predict whether this has any effect on post-treatment parasite transmission, it is essential to repeat our study with *Plasmodium*-infected participants. Further, it is recommended that these experiments are repeated with other commonly used ACT medicines, particularly those that have limited gametocytocidal effects. For example, a recent study reported that the appearance of gametocytaemia after ACT-treatment was higher when artesunate/amodiaquine and dihydroartemisinin–piperaquine were used compared to AL [14]. No effect of AL on fitness parameters of *An. coluzzii* was found. Based on these results, it appears unlikely that AL alone has an effect on post-treatment transmission through direct or indirect effects on *Anopheles* mosquitoes.

## Additional file

**Additional file 1: Table S1.** Pairwise comparison (LSD) of mosquito response to skin odour of eight participants in the olfactometer assay. Values in bold indicate significant differences.

**Additional file 2: Table S2.** Data generated and analysed in this study, including raw data of the olfactometer experiment, summary of the olfactometer data, screenhouse experiment data and data of the fitness experiment.
