# Supplementary material for: Does artemether–lumefantrine administration affect mosquito olfactory behaviour and fitness?
Source: Malar J. 2019 Jan 28;18:28. doi: 10.1186/s12936-019-2646-9 (PMC6350316; doi:10.1186/s12936-019-2646-9)
Supplement: Supplementary file 1 — Additional file 1: Table S1. Pairwise comparison (LSD) of mosquito response to skin odour of eight participants in the olfactometer assay. Values in bold indicate significant differences. [file 12936_2019_2646_MOESM1_ESM.docx]

**Additional file 1:**

Main article: Does artemether-lumefantrine administration affect mosquito olfactory behaviour and fitness?

Authors: Jetske G. de Boer, Annette O. Busula, Jet ten Berge, Tessa S. van Dijk, Willem Takken

Description of additional file 1: pairwise comparison of mosquito response to odour samples of individual participants (Table S1).

Table S1: Pairwise comparison (LSD) of mosquito response to skin odour of eight participants in the olfactometer assay. Values in bold indicate significant differences.

| Participant | 1 | 2 | 3 | 4 | 7 | 8 | 9 | 10 |
| --- | --- | --- | --- | --- | --- | --- | --- | --- |
| 1 |  |  |  |  |  |  |  |  |
| 2 | 0.172^a^ |  |  |  |  |  |  |  |
| 3 | **0.001** | 0.089 |  |  |  |  |  |  |
| 4 | 0.774 | 0.281 | **0.004** |  |  |  |  |  |
| 7 | 0.733 | 0.308 | **0.005** | 0.956 |  |  |  |  |
| 8 | 0.525 | 0.461 | **0.012** | 0.728 | 0.771 |  |  |  |
| 9 | 0.468 | 0.52 | **0.016** | 0.661 | 0.703 | 0.926 |  |  |
| 10 | **0.028** | 0.438 | 0.35 | 0.057 | 0.066 | 0.121 | 0.147 |  |

^a)^ P-values for pairwise comparison, following GLM with number of mosquitoes attracted to the skin odour samples as response variable and total number of mosquitoes that flew in the olfactometer as binomial total. Mosquito numbers were summed over five replicates. GLM included sampling timepoint (P = 0.026) and participant identity (P = 0.033) as explanatory variables.
